# Digital Health Interventions for Family Members of ICU Patients: Current Evidence and Future Directions

**DOI:** 10.2196/95876

**Published:** 2026-04-30

**Authors:** Tanya Abenaim, Dan Poenaru, Michael Goldfarb

**Affiliations:** 1 Faculty of Medicine and Health Sciences, McGill University, Montreal, QC, Canada; 2 Division of Pediatric Surgery, Montreal Children's Hospital, Montreal, QC, Canada; 3 Division of Cardiology, Jewish General Hospital, McGill University, Office E-212, 3755 Cote Ste Catherine Road, Montreal, QC, H3T 1E2, Canada, 1 514-340-8222

**Keywords:** family engagement, digital health interventions, intensive care units, post-intensive care syndrome-family, PICS-F

## Abstract

A recent systematic review and meta-analysis of randomized trials evaluating digital health interventions for family members of intensive care unit (ICU) patients found no significant improvements in anxiety, depression, posttraumatic stress, quality of life, or communication quality. Rather than concluding that digital approaches are inherently ineffective, we argue that these null findings reflect identifiable and remediable limitations in intervention design, outcome measurement, and trial methodology. In this commentary, we examine four structural barriers that currently constrain the evidence base and outline the conditions that next-generation trials must meet to adequately address the questions raised by this review.

Intensive care admissions rarely affect only the patient. Family members of critically ill patients also carry a substantial and well-documented psychological burden. Post–intensive care syndrome–family (PICS-F) affects a significant proportion of relatives of patients in the intensive care unit (ICU), with symptoms such as anxiety, depression, and posttraumatic stress that can persist well beyond ICU discharge. Distressed family members may be less able to provide the emotional and practical support that facilitates patient recovery, making PICS-F not only a humanitarian concern but also an important factor in patient-outcomes.

Against this backdrop, digital health interventions (DHIs) have attracted substantial interest as scalable solutions—encompassing mobile apps, web-based platforms, videoconferencing, virtual reality, and internet-delivered cognitive behavioral therapy. Zhang and colleagues [1] have now published a systematic review with meta-analysis of randomized controlled trials (RCTs) evaluating DHIs specifically for family members of ICU patients, synthesizing 17 trials and 1864 participants across three continents. No statistically significant effects were found for any of the primary outcomes: anxiety, depression, posttraumatic stress disorder (PTSD), quality of life, or communication quality. Wide prediction intervals and substantial statistical heterogeneity indicated marked variability in effects across settings. However, those intervals warrant closer examination than a simple null verdict would suggest.

## Interpreting the Null Result

A null meta-analytic result is neither proof of no effect nor license for the field to continue unchanged. Both interpretations remain plausible: that DHIs are conceptually sound but poorly executed and that they are genuinely ineffective for this population. The available evidence cannot adjudicate between them. What it does allow is a clearer diagnosis of why the evidence remains uninformative.

The 95% prediction interval for anxiety (−1.46 to 0.79) is the most consequential figure in the paper. Its upper boundary indicates the possibility of clinically meaningful harm. However, Zhang et al [1] report that no adverse outcomes were prospectively monitored across any of the 17 included trials—a striking omission given that all intervened in a population already at elevated risk for PTSD, depression, and complicated grief. The same adverse-event reporting standards applied to pharmaceutical RCTs should apply here. An undetected signal cannot be assumed benign; it may be neutral, heterogeneous across subgroups, or—in a subset of vulnerable families—harmful.

The heterogeneity is not merely statistical. A 14-minute virtual reality exposure [2] is being pooled with a 5-week internet-based cognitive behavioral writing program [3]. These are fundamentally different interventions, illustrating the absence of a coherent intervention taxonomy. This is not a criticism of the meta-analysis itself, but a call for theory-driven subgroup classification before the next generation of trials is designed.

## Four Structural Barriers to Conclusive Evidence

Taken together, these issues point to four structural barriers that currently prevent the field from generating conclusive evidence.

### Barrier 1: Underdeveloped Intervention Theory

Most included DHIs were information-centric: unidirectional, static, and content-driven. Qualitative evidence consistently attributes PICS-F persistence to uncertainty, helplessness, traumatic witnessing, and disrupted attachment [4]—psychological processes for which passive information delivery is likely insufficient. The gap between what families experience and what current DHIs address is not incidental; it is a design failure that must be corrected before outcome data can be meaningfully interpreted.

### Barrier 2: Mismatch Between Measurement and mechanism

Before the next generation of trials is designed, the field requires a clearer account of what DHIs are hypothesized to change and through what pathway. For instance, *reduced anxiety at 12 weeks* is an outcome, not a mechanism; it cannot distinguish whether an intervention succeeded by reducing informational uncertainty, increasing perceived control, facilitating emotional processing, or simply providing structured human contact through a digital medium. Without mechanistic specificity, outcome selection remains arbitrary and follow-up windows cannot be meaningfully calibrated.

PTSD is a delayed-onset condition; its prevention requires follow-up windows matched to its natural history, not 3 months or fewer that most included studies employed. Notably, several trials reported positive subjective family feedback despite nonsignificant quantitative outcomes, a pattern Zhang et al interpret as suggesting that current instruments may lack the sensitivity to capture experiential gains that families themselves perceive. If families are experiencing meaningful benefit that validated scales cannot detect, that is a measurement failure as much as an intervention failure, and it strengthens the case for multimodal outcome assessment, including patient- and family-reported experience measures alongside standardized psychological instruments.

### Barrier 3: Near-Total Absence of Engagement Data

Petrinec et al [5] reported a mean of 11.4 application logins and 50 minutes of total use across a study period, a dose unlikely to produce meaningful behavioral change. Whether current DHIs fall short because they are conceptually inadequate or simply not used enough cannot be determined from outcomes data alone. Answering this question requires dose-response analyses and engagement-stratified outcome reporting, neither of which was conducted in the included trials.

### Barrier 4: Outcome Measure Heterogeneity

Across 17 included studies, Zhang et al identified 18 distinct validated instruments, rendering cross-trial comparisons interpretively fragile. However, no PICS-F-specific core outcome set initiative has yet been registered with Core Outcome Measures in Effectiveness Trials (COMET). developing one should be a methodological priority [6].

## Requirements for the Next Generation of Trials

Future trials require interventions grounded in behavioral theory, delivered longitudinally, with pre-specified engagement thresholds, prospective adverse-event monitoring, and follow-up windows matched to the outcomes being targeted. Equity must be a design requirement, not an afterthought: older adults, non-English-speaking families, and low-income caregivers bear a disproportionate PICS-F burden yet face the greatest barriers to DHI access [7]. If equity considerations are not embedded in eligibility criteria and delivery design, effective interventions will reach only the most resourced families, deepening the disparities they might otherwise address.

Table 1 traces the evolution of DHI design toward a third generation defined by artificial intelligence (AI)–driven personalization, adaptive delivery, and bidirectional clinician–family communication. These tools represent a genuine conceptual advance over what has been tested—but not yet a proven one. The Society of Critical Care Medicine has articulated the case for family-centered ICU care [8]; building the evidence base to deliver it is the work that remains.

**Table 1.** Evolution of digital health intervention design for families of ICU patients.

Generation	Characteristics	Examples	Limitations
First generation	Static information delivery One-way communication	Educational websites SMS updates	Low engagement Generic content
Second generation	Interactive platforms Basic personalization	Mobile apps with feedback forms Video calls	Limited artificial intelligence (AI) integration Reactive rather than proactive
Third generation (emerging)	AI–driven personalization Predictive analytics Real-time adaptation Culturally adaptive content Integration with clinical workflows	Chatbots with natural language processing Adaptive content delivery	Nascent evidence base Requires rigorous evaluation before adoption

What Zhang et al actually demonstrate is not that DHIs for ICU families do not work. It is that we do not yet know whether they work, in whom, at what dose, or through what mechanism. Answering these questions will require a fundamentally different approach to trial design than the field has so far adopted.

## Abbreviations

AI: aritfiical intelligence
DHI: digital health intervention
ICU: intensive care unit
PICS-F: post–intensive care syndrome–family
PTSD: posttraumatic stress disorder
RCT: randomized controlled trial
